# Obstructive shock in pediatric patient with congenital cystic adenomatoid malformation: A case report

**DOI:** 10.1016/j.amsu.2021.102614

**Published:** 2021-07-28

**Authors:** Puspita Ayu Margaretha, Desy Rusmawatiningtyas, Firdian Makrufardi, Intan Fatah Kumara, Nurnaningsih

**Affiliations:** Department of Child Health, Faculty of Medicine, Public Health and Nursing, Universitas Gadjah Mada/Dr. Sardjito Hospital, Yogyakarta, 55281, Indonesia

**Keywords:** Obstructive shock, Congenital anomaly, Adenomatoid malformation, Pediatric, Case report

## Abstract

**Introduction:**

and importance: Congenital cystic adenomatoid malformation (CCAM) is a rare cystic lesion in the lungs. CCAM might present in the early neonatal period with symptoms of respiratory distress.

**Case presentation:**

A 2-year-old girl was admitted to our Pediatric Intensive Care Unit with signs of severe respiratory distress. She had been diagnosed with CCAM since she was three months old. She also had undergone several procedures such as thoracotomy and decortication since then, but she still suffered some episodes of pulmonary infection. In this admission, her computerized thoracic tomography revealed a pleuropulmonary blastoma (PPB).

**Clinical discussion:**

An obstruction of blood outflow from the left ventricle could happen when an intrathoracic mass exists, leading to a decrease of cardiac output and resulting in an obstructive shock, which could be fatal. One of the malignancy types commonly occurring is pleuropulmonary blastoma (PPB), which has a poor prognosis. Early detection on CCAM can be done by prenatal ultrasound.

**Conclusion:**

Obstructive shock is one of complication that might occur in pleuropulmonary blastoma.

## Highlights

• Pleuropulmonary blastoma (PPB) has a poor prognosis.
• Lobectomy is recommended to prevent recurrence of infection.
• Early detection on CCAM could be done by prenatal ultrasound.

## 1 Introduction

Congenital cystic adenomatoid malformation (CCAM) is a rare cystic lesion in the lungs with an incidence of one in 11,000 to 35,000 births [1]. CCAM might present in the early neonatal period with symptoms of respiratory distress [2]. Previous reports showed that 86 % of asymptomatic patients will be symptomatic at two years old [[1], [2], [3]]. The definitive therapy of CCAM is surgery. Lobectomy is correlated to low mortality and morbidity and could prevent complications such as infection and transformation to malignancy. With lobectomy, the lung function will usually be resolved, resulting in normal total lung volume. Research showed CCAM could be resolved completely with only 4 % that needed further interventions [5].

One of 8 patients with CCAM will develop pleuropulmonary blastoma (PPB) [5]. A study mentioned that 31 % of PPB appeared in patients with CCAM [4]. Prognosis of CCAM patients with PPB is considered poor. Half of the patients were reported deceased one year after being diagnosed [4,6]. The worst prognosis might be predicted from tumor size >5 cm and metastasis at the first time of diagnosis [7]. From 1600 patients admitted to the Intensive Care Unit, 2 % presented with obstructive shock due to various etiology [8]. Intrathoracic mass was one of its causes which results in decreasing of cardiac output [9]. This case was reported in line with the Surgical CAse REport (SCARE) criteria [10].

## 2 Case presentation

The patient was born from a Gravida 4 Para 3 mother, at 39 weeks gestational age from spontaneous delivery with birth weight 3600 g. Her mother never had an ultrasound during the pregnancy. History of basic and booster immunization was complete. The first bullectomy was done when she was four months old. She was then discharged from the hospital in good condition. When she was eight months old, she developed a fever and had difficulty of breathing. The physical examination showed tachypnea, chest wall retraction, and decreased vesicular sounds at the left lung. The thorax imaging showed pulmonary abscess at the left lung and thoracotomy and decortication were executed at previous hospital.

At one year old, the patient had a similar complaint. Computerized tomography (CT)-scan imaging showed CCAM type 1 in the left lung's inferior lobe that deviated the mediastinum to the right, and again thoracotomy and bullectomy were executed. Three months later, the patient presented again with fever, coughing and shortness of breath.

**Fig. 1:**
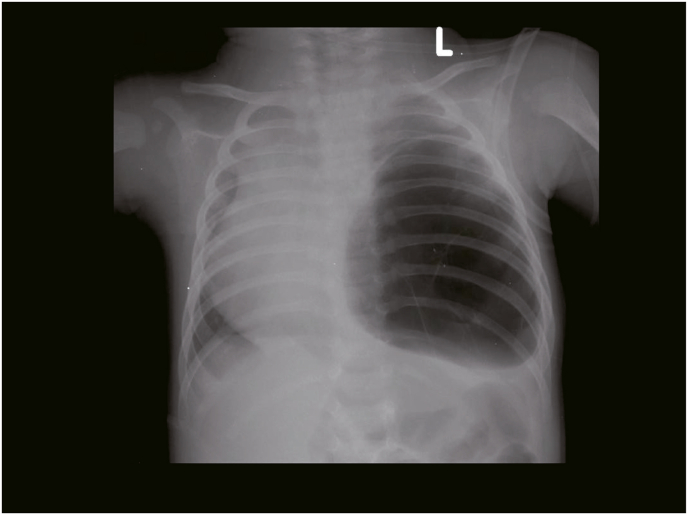
Thorax imaging showed a figure of heterogenous mass (solid-cystic) in the left hemithorax.

**Fig. 2:**
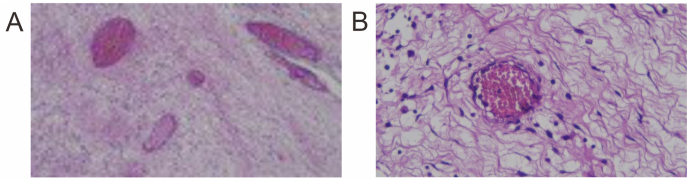
(A) Connective tissue in the form of spaces lined with cuboid epithelium; (B) Histopathology of congenital cystic adenomatoid malformation (CCAM).

At the age of two years old, she presented with another set of similar complaints. The physical examination showed tachypnea, chest wall retraction, rhonchi on both lungs, with decreased vesicular sounds at the left lung. Thorax imaging (Fig. 1) showed a figure of heterogenous mass (solid-cystic) in the left hemithorax that shifted the mediastinal structure to the right, which led to the PPB type 2 with CCAM diagnoses. Partial lobectomy was done and the pathology/anatomy examinations found CCAM presentation (Fig. 2) with chronic suppurative inflammation. A lobectomy was not done in the earlier surgeries, which may have caused recurrences in this patient. The patient chose to discharge against medical advice.

**Fig. 3:**
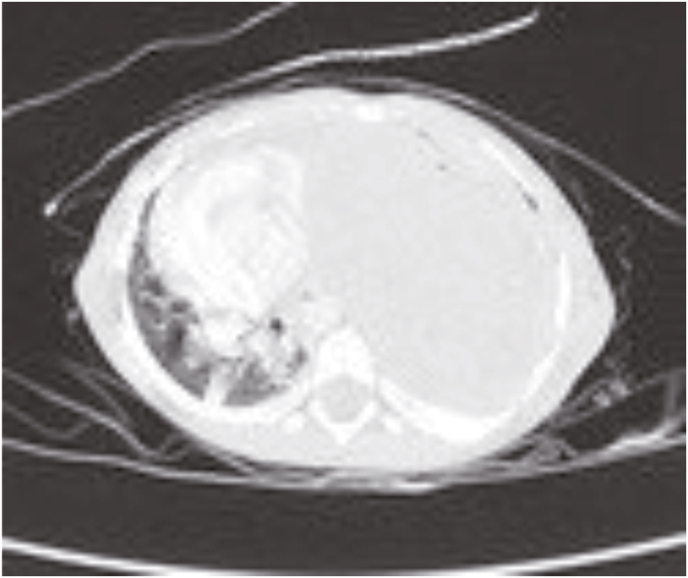
Pleuropulmonary blastoma type 2 (mixed) caused the mediastinum to shift to the right in thorax CT-scan.

Five months later, the patient was admitted to our hospital again with recurrent pneumonia. During the hospitalization, the patient had fever and shortness of breath. The physical examination showed tachypnea, chest wall retraction, with decreased vesicular sounds at the left lung, followed by rhonchi and crepitation. From x-ray imaging, a round opacity was found in the left hemithorax which caused the mediastinum to shift to the right, leading to pulmonary abscess appearance and bilateral pneumonia. Thorax CT-scan results (Fig. 3) showed PPB type 2 (mixed) appearance in the entire inferior and superior lingular lobe of the left lung which caused the mediastinum shift to the right resulting in the collapse of the apicoposterior segment of the superior lobe in the left lung. The lesion size became 9.45 x 11.38 × 11.55 cm from the previous size of 8.7 x 9.5 × 10.3 cm.

On the 8th day of treatment, she experienced cardiorespiratory failure and the blood gas analysis results indicated a very severe respiratory acidosis so immediately the patient was intubated. Laboratory results showed an elevated procalcitonin level up to >200 ng/mL, glomerulus filtration rate was decreased to 42 with creatinine at 0.85 mg/dL. The blood culture found *Staphylococcus hominis*. Antibiotic treatment was escalated. She never had a cardiac evaluation. The patient had tachycardia and decreased peripheral perfusion that did not improve after fluid resuscitation. We began dopamine therapy, but she still had tachycardia, hypotension, decreasing peripheral perfusion, and oliguria. Dobutamine and norepinephrine were given without any response to the therapy. Due to her condition, a lobectomy was not done in this admission.

## 3 Discussion

Here, we reported a case of a 2-year-old female patient with CCAM in our tertiary hospital. Obstructive shock is a condition caused by either big vessel or cardiac ventricle obstruction [15]. The mechanical factor of intravascular or extravascular pressure could decrease the blood flow in big vessels or blood outflow, leading to a decrease in cardiac output and oxygen delivery, resulting in tissue hypoxia [16]. There are several cases that can lead to obstructive shock, such as tension pneumothorax, cardiac tamponade, pulmonary embolism, and left sided cardiac obstruction [15]. In one case, a young male with mediastinal mass experienced obstructive shock [17]. The mass that shifts the mediastinal structure to the right causes an obstruction of blood outflow from the left ventricle decreasing cardiac output and resulting in low oxygen perfusion to the target tissue [9,18]. This could lead to an imbalance in oxygen delivery and oxygen consumption. Treatment for this type of obstructive shock is to relieve the obstruction [16]. In this patient, we found a 9 cm diameter tumor, which could not be removed and rapidly progressed to obstructive shock. If the obstruction can not be excluded, the decompensated shock would occur faster due to the hypotension stage that would not respond to inotropic intervention and could potentially cause death [19].

CCAM is typically diagnosed by prenatal ultrasonography (USG) from the 12th to 18th gestational week [20]. The World Health Organization (WHO) recommends to do ultrasound scan before 24 weeks of gestation to improve detection of fetal anomalies, but due to low socioeconomic status of this patient, her mother never had the fetal ultrasound [21]. This is a very common condition in Indonesia where prenatal ultrasound is not done routinely due to financial problems. In CCAM suspected cases, there is a high-resolution fetal ultrasound to evaluate fetal anatomy and monitor any lung lesion. Depending on the size of the CCAM, ultrasound surveillance should be performed once or twice a week through mid-gestation to monitor the volume changes of the CCAM [21,22]. Once a lung mass is identified on ultrasound, color doppler and fetal echocardiography should be done. In the case of a baby with a large CCAM that continues to grow but hydrops does not develop, a special delivery technique called ex-utero intrapartum treatment (EXIT) procedure may be required [22].

A previous study recommended magnetic resonance imagery (MRI) to aid in the diagnosis of atypical patients with multiple anomalies in the prenatal period [21]. The presence of fetal hydrops and cysts >5 mm with a solid appearance denote poor prognosis. Hemodynamic changes due to cardiac compression have been shown to cause hydrops. Despite the ability to identify anomalies using USG, thoracic radiography of most newborns with CCAM is normal. In the postnatal period, lesions are easily found on CT scan imagery. Through imaging modalities, abnormal air is observed in CCAM type 1, air-fluid level in type 2, and fluid-filled cysts with solid structure in type 3. Definitive diagnosis, however, can be established through histopathological evaluation [11].

The definitive therapy of CCAM is surgery [13]. With a lobectomy, the remaining lung could develop, thus, the total lung volume and its function will improve to normal levels [14]. The lobectomy procedure has a low rate of complications, and this intervention is expected to prevent recurrent lung infection and its transformation to malignancy [5]. From thorax CT-scan imaging, in our patient, the mediastinal structure was shown shifting to the right, causing a very severe respiratory acidosis for the patient due to the air trapping that led to carbon dioxide elevation.

PPB is usually present in children younger than five years old [4]. PPB is an aggressive malignancy that originated from the lung, mediastinum, diaphragm, or pleura. PPB which is classified as type 1 is usually diagnosed at ten months old with cystic lesion appearance, type 2 when cystic and solid lesion is found, and type 3 is a solid lesion appearance [12].

The prognosis of PPB patients is poor; half of the patients were reported deceased one year after diagnosis [4,6]. A study revealed that the worst prognosis might be predicted from tumor size >5 cm and the presence of metastasis when first time diagnosed [7]. PPB type II and III have a 62 % survival number until two years old and 42 % until five years old. Patients with pleura, mediastinal, or extrapulmonary involvement since first diagnosed have the worst prognosis [14]. In this patient, we found PPB type II appearance with the lesion size of 9.08 x 9.07 × 10.67 cm. This patient died due to obstructive shock.

## 4 Conclusions

Congenital cystic adenomatoid malformation (CCAM) in children may have symptoms such as acute respiratory distress and/or a recurrent lung infection. Obstructive shock is one of complication that might occur in pleuropulmonary blastoma. Moreover, we recommend detection of fetal anomalies in the prenatal period to detect possible CCAM and prevent complications.

## Annals of medicine and surgery

The following information is required for submission. Please note that failure to respond to these questions/statements will mean your submission will be returned. If you have nothing to declare in any of these categories then this should be stated.

## Please state any sources of funding for your research

The authors declare that this study had no funding source.

## Ethical approval

The informed consent form was declared that patient data or samples will be used for educational or research purposes. Our institutional review board also do not provide an ethical approval in the form of case report.

## Consent

Written informed consent was obtained from the patient for publication of this case report and accompanying images. A copy of the written consent is available for review by the Editor-in-Chief of this journal on request.

## Author contribution

Puspita Ayu Margaretha, Desy Rusmawatiningtyas, Nurnaningsih conceived the study and approved the final draft. Puspita Ayu Margaretha, Desy Rusmawatiningtyas, Firdian Makrufardi, Intan Fatah Kumara, Nurnaningsih drafted the manuscript, and critically revised the manuscript for important intellectual content. Puspita Ayu Margaretha, Desy Rusmawatiningtyas, Firdian Makrufardi, Intan Fatah Kumara, Nurnaningsih facilitated all project-related tasks.

## Registration of research studies

This is not a ‘first in humans’ report, so it is not in need of registration.

## Guarantor

Desy Rusmawatiningtyas.

## Funding source

This research did not receive any specific grant from funding agencies in the public, commercial, or not-for-profit sectors.

## Provenance and peer review

Not commissioned, externally peer-reviewed.

## Declaration of competing interest

No potential conflict of interest relevant to this article was reported.
